# Incidence trends of childhood central nervous system tumors in Finland 1990–2017

**DOI:** 10.1186/s12885-022-09862-0

**Published:** 2022-07-18

**Authors:** Jad Abuhamed, Atte Nikkilä, Jani Raitanen, Wafa Alimam, Olli Lohi, Janne Pitkäniemi, Hannu Haapasalo, Anssi Auvinen

**Affiliations:** 1 The Health Sciences Unit, Faculty of Social Sciences, Tampere University, Arvo Ylpön katu 34, 33520 Tampere, Finland; 2 TamCAM - Tampere Center for Child, Adolescent and Maternal Health Research, Tampere University, Tampere, Finland; 3 UKK Institute for Health Promotion Research, Tampere, Finland; 4 Department of Pediatrics and Tays Cancer Center, Tampere University Hospital, Tampere, Finland; 5 Finnish Cancer Registry, Institute for Statistical and Epidemiological Cancer Research, Helsinki, Finland; 6 Department of Public Health, Faculty of Medicine, University of Helsinki, Helsinki, Finland; 7 Fimlab Laboratories, Tampere, Finland; 8 STUK – Radiation and Nuclear Safety Authority, Helsinki, Finland; 9 Tampere University Hospital, Tampere, Finland

**Keywords:** Incidence, CNS tumors, Childhood cancer, Trends, Cancer register

## Abstract

**Introduction:**

Central nervous system (CNS) tumors are a leading cause of cancer-related morbidity and mortality in children. Our aim is to characterize incidence trends of pediatric CNS tumors in Finland over the last three decades.

**Methods:**

Data on all benign and malignant incident CNS tumors diagnosed in children aged 0–14 years in 1990–2017 were extracted from the Finnish Cancer Registry and classified according to the 2016 WHO classification of CNS tumors. We analyzed age-standardized incidence rates (ASR) for pediatric CNS tumors overall and by sex, age, tumor histology, grade, and location using Poisson regression. We used joinpoint regression to evaluate changes in trends.

**Results:**

Overall, 1117 pediatric CNS tumor cases were registered in Finland with a 1.2:1 male to female ratio. The average annual ASR was 4.3 per 100,000 person-years (95% CI 4.26, 4.34). The most common tumor type was pilocytic astrocytoma (30% of tumors), followed by medulloblastoma (10%) with incidence rates of 1.30 and 0.45 per 100,000 person-years, respectively. The overall incidence of pediatric CNS tumors increased by an annual percentage change (APC) of 0.8% (95% CI 0.2, 1.4). We observed no major changes in incidence trends of tumor histology groups or tumor location groups. The ASR of benign tumors increased by an APC of 1.0 (95% CI 0.1, 2.0).

**Conclusions:**

Utilizing the high-quality and completeness of data in the Finnish Cancer registry, we found that the incidence of pediatric CNS tumors in Finland has increased slightly from 1990 until 2017. Although variations in diagnostic and registration practices over time might have affected the rates, the trend may also reflect a true increase in incidence.

## Introduction

Central nervous system (CNS) tumors are the second most common group of childhood neoplastic diseases after leukemia and a leading cause of cancer-related death among children [1]. The global incidence of CNS tumors in children is estimated at 2.8 per 100,000 person-years [1]. These tumors constitute a heterogeneous group of pathologic entities with different biology, and their incidence, histologic type, and prognosis in children are distinct from those in adults [2]. Between the 1970s and 1990s, the incidence of pediatric CNS tumors increased in several European countries and the United States [3, 4]. Recent studies from Canada and France reported stable incidence rates of pediatric CNS tumors, while a study from the US showed a small but significant increase [5–7]. As the etiology of CNS tumors is still largely unknown, monitoring changes in cancer incidence is critical for instigating hypothesis-based research on potential environmental risk factors, as well as assessing the public health burden [8].

However, the characterization of CNS tumor incidence and temporal trends involves several challenges. The coding and classification of CNS tumors have been amended and updated over the years, reflecting the evolving pathological and clinical knowledge as showcased lately by the incorporation of molecular parameters into the 2016 WHO classification of CNS tumors [9]. Another example is downgrading the tumor behavior of pilocytic astrocytoma from malignant to uncertain in the 2000 edition of ICD-O-3 [10]. Moreover, cancer registers vary in completeness and inclusion of benign CNS tumors, which can be premalignant and potentially life-threatening. In the US, registration of benign CNS tumors was mandated by law in 2004 compared to 1953 in Finland [11, 12]. The completeness of the Finnish Cancer Registry (FCR) for childhood tumors was estimated as 94% for 2009–2013 [13].

In this study, we evaluate incidence trends of childhood CNS tumors during 1990–2017 by sex, age, tumor histology, grade, and location utilizing the population-based high-quality data of benign and malignant childhood tumors in Finland based on the 2016 WHO classification of CNS tumors.

## Materials and methods

Data on all benign and malignant CNS tumors cases registered in children aged 0–14 years between 1990 and 2017 were extracted from the FCR based on the topographical categories: brain, meninges, and central nervous system (ICD-10 codes C70–72, D32–33, and D42–43). We included only first primary cancers. CNS lymphomas were excluded. Tumors were coded using ICD-O-3 (first and second revision) and grouped by the 2016 WHO classification of central nervous system tumors [9] into six histology groups (Table 1): 1) diffuse astrocytic and oligodendroglial tumors, 2) other astrocytic tumors (low-grade gliomas), 3) ependymal tumors, 4) neuronal and mixed neuronal-glial tumors, 5) embryonal tumors, and 6) other tumors which included choroid plexus tumors, other gliomas, tumors of the cranial and paraspinal nerves, meningiomas, mesenchymal tumors, melanocytic tumors, germ cell tumors, malignant glioma not otherwise specified (NOS), and unclassified tumors.

**Table 1:** Modified classification of the central nervous system (CNS) tumors based on the 2016 World Health Organization Classification of Tumors of the CNS [9]

Tumor group	Morphology code
(1) Diffuse astrocytic and oligodendroglial tumors	
Diffuse astrocytomas grade II-III	9400, 9401
Oligodendrogliomas, grade II-III	9450, 9451
Mixed oligo-astrocytoma, grade II-III	9382
Glioblastoma	9440, 9441
Diffuse midline glioma, H3 K27M–mutant	9385
Gliomatosis cerebri (growth pattern)	9381
(2) Other astrocytic tumors (low-grade gliomas)	
Pilocytic astrocytoma	9421
Pilomyxoid astrocytoma	9425
Subependymal giant-cell astrocytoma	9384
Pleomorphic xanthoastrocytoma	9424
(3) Ependymal tumors	
Subependymoma	9383
Ependymomas grade II-III	9391, 9392, 9394
Ependymoma, RELA fusion-positive	9396
(4) Neuronal and mixed neuronal-glial tumors	
Dysembryoplastic neuroepithelial tumor	9413
Gangliocytoma	9492
Ganglioglioma	9505
Dysplastic gangliocytoma of cerebellum	9493
Desmoplastic infantile ganglioglioma	9412
Central neurocytoma	9506
(5) Embryonal tumors	
Medulloblastoma	9470, 9471, 9474
Central neuroblastoma	9500
Ganglioneuroblastoma	9490
CNS embryonal tumor NOS	9473
Atypical teratoid/rhabdoid tumor	9508
(6) Other tumors	
Choroid plexus tumors	9390
Other gliomas	9431, 9430, 9423
Tumors of the cranial and paraspinal nerves	9560, 9540, 9550
Meningiomas	9530, 9537, 9531, 9538, 9539
Mesenchymal, non-meningothelial tumors	9120, 9364, 9150, 8800, 8963, 8990
Melanocytic tumors	8728
Germ cell tumors	9064, 9080, 9084, 9081
Malignant glioma NOS	9380
Unclassified tumors	8000, 8982

The following morphology codes were appended to the 2016 WHO classification: 9381 (gliomatosis cerebri, growth pattern); 9423 (polar spongioblastoma); 9380 (malignant glioma NOS); 8800, 8963, 8990 (other sarcomas); and 9081 (teratocarcinoma). We excluded ten cases of recurrent tumors from the study. The population size was retrieved from Statistics Finland by single-year age and sex for each year of the study period [14]. The mean annual population size was 924,605 for children aged 0–14 years. Incidence rates were age-standardized using the 2013 European standard population and calculated per 100,000 person-years stratified by sex, age group, tumor histology, tumor grade, and tumor location [15].

The differences in incidence rates between the groups were estimated by incidence rate ratios (IRR). We used joinpoint regression to evaluate changes in incidence trends as annual percentage change (APC) (Joinpoint Regression Program, Version 4.7.0.0. February 2019; Statistical Research and Applications Branch, National Cancer Institute). Stata software was used for other analyses (StataCorp. 2019. Stata Statistical Software: Release 16. College Station, TX: StataCorp LLC). *P*-values below 0.05 were considered statistically significant, and all tests were two-tailed.

## Results

During 1990–2017, 1117 pediatric CNS tumors were registered in Finland, 614 in boys and 503 in girls (Table 2). Tumors were most frequent in the age group 0–4 years (428 tumors). The predominant tumor location was the infratentorial area with an age-standardized incidence rate (ASR) of 1.80 (95% CI 1.77, 1.83) per 100,000 person-years, followed by the supratentorial brain (ASR 1.34, 95% CI 1.32, 1.37), and the spinal cord (ASR 0.25, 95% CI 0.24, 0.26). Tumors registered with an unspecified location comprised 19% of all tumors. Overall, 96% of the tumors were histologically verified. Gliomas accounted for half of the tumors, with low-grade gliomas comprising the largest tumor group in the study. Pilocytic astrocytoma was the most common tumor (ASR 1.30, 95% CI 1.28, 1.33) followed by medulloblastoma (ASR 0.45, 95% CI 0.43, 0.46), constituting 30 and 10% of all tumors, respectively. Most embryonal tumors and low-grade gliomas were infratentorial (71 and 52%, respectively). Benign tumors (grade I) comprised 48% of the tumors (ASR 2.05, 95% CI 2.02, 2.08), and highly malignant (grade IV) comprised 22% (ASR 0.95, 95% CI 0.93, 0.97). Overall, 32% of the infratentorial and 17% of supratentorial tumors were grade IV tumors. Of the 66 tumors in the spinal cord, 18% were pilocytic astrocytomas, and 12% ependymomas.

**Table 2:** Age-standardized incidence rates (ASR) per 100,000 person-years with 95% confidence intervals (CIs) of pediatric CNS tumors in Finland 1990–2017

	Total		Boys		Girls	
	N (%)	ASR (95% CI)	N (%)	ASR (95% CI)	N (%)	ASR (95% CI)
**Total**	1117 (100)	4.30 (4.26, 4.34)	614 (100)	4.63 (4.57, 4.69)	503 (100)	3.96 (3.91, 4.02)
**Age group**						
0–4 years	428 (38)	5.08 (4.60, 5.56)	242 (39)	5.62 (4.91, 6.33)	186 (37)	4.51 (3.86, 5.16)
5–9 years	363 (33)	4.20 (3.77, 4.63)	200 (33)	4.53 (3.90, 5.16)	163 (32)	3.85 (3.26, 4.45)
10–14 years	326 (29)	3.70 (3.30, 4.10)	172 (28)	3.82 (3.25, 4.39)	154 (31)	3.57 (3.01, 4.13)
**Tumor grade**						
Grade I	531 (48)	2.05 (2.02, 2.08)	285 (46)	2.15 (2.11, 2.19)	246 (49)	1.94 (1.90, 1.98)
Grade II	155 (14)	0.60 (0.58, 0.61)	80 (13)	0.60 (0.58, 0.62)	75 (15)	0.59 (0.57, 0.61)
Grade III	99 (9)	0.38 (0.37, 0.39)	53 (9)	0.40 (0.38, 0.41)	46 (9)	0.36 (0.35, 0.38)
Grade IV	246 (22)	0.95 (0.93, 0.97)	152 (25)	1.14 (1.11, 1.17)	94 (19)	0.74 (0.72, 0.77)
Grade NA	86 (8)	0.33 (0.32, 0.34)	44 (7)	0.33 (0.31, 0.34)	42 (8)	0.33 (0.31, 0.34)
**Tumor location**						
Supratentorial	349 (31)	1.34 (1.32, 1.37)	178 (29)	1.34 (1.31, 1.38)	171 (34)	1.35 (1.31, 1.38)
Infratentorial	468 (42)	1.80 (1.77, 1.83)	262 (43)	1.97 (1.93, 2.01)	206 (41)	1.62 (1.59, 1.66)
Cerebellum	324 (29)	1.24 (1.22, 1.27)	185 (30)	1.39 (1.36, 1.42)	139 (28)	1.09 (1.06, 1.12)
Brain stem	138 (12)	0.53 (0.52, 0.55)	77 (13)	0.58 (0.56, 0.60)	61 (12)	0.48 (0.46, 0.50)
Spinal cord	66 (6)	0.25 (0.24, 0.26)	42 (7)	0.32 (0.30, 0.33)	24 (5)	0.19 (0.18, 0.20)
Meninges	26 (2)	0.10 (0.09, 0.11)	15 (2)	0.11 (0.10, 0.12)	11 (2)	0.09 (0.08, 0.10)
Unspecified	208 (19)	0.80 (0.78, 0.82)	117 (19)	0.88 (0.85, 0.91)	91 (18)	0.72 (0.69, 0.74)
**Histology**						
Diffuse astrocytic tumors	161 (14)	0.62 (0.61, 0.64)	72 (12)	0.55 (0.52, 0.57)	89 (18)	0.71 (0.68, 0.73)
Other astrocytic tumors	374 (33)	1.44 (1.42, 1.46)	192 (31)	1.45 (1.42, 1.48)	182 (36)	1.43 (1.40, 1.47)
Pilocytic astrocytoma	339 (30)	1.30 (1.28, 1.33)	176 (29)	1.33 (1.30, 1.36)	163 (32)	1.28 (1.25, 1.31)
Ependymal tumors	85 (8)	0.33 (0.31, 0.34)	54 (9)	0.40 (0.39, 0.42)	31 (6)	0.24 (0.23, 0.26)
Neuronal/glial tumors	118 (11)	0.46 (0.44, 0.47)	65 (11)	0.49 (0.47, 0.51)	53 (11)	0.42 (0.40, 0.44)
Embryonal tumors	190 (17)	0.73 (0.71, 0.74)	123 (20)	0.92 (0.89, 0.95)	67 (13)	0.53 (0.51, 0.55)
Medulloblastoma	116 (10)	0.45 (0.43, 0.46)	74 (12)	0.56 (0.54, 0.58)	42 (8)	0.33 (0.32, 0.35)
Other tumors	189 (17)	0.73 (0.71, 0.74)	108 (18)	0.81 (0.79, 0.84)	81 (16)	0.64 (0.61, 0.66)

The average annual ASR of all pediatric CNS tumors combined was 4.30 (95% CI 4.26, 4.34) per 100,000 person-years (Table 2). The ASR in boys was higher than in girls (IRR = 1.17, 95% CI 1.04, 1.32) with a 1.2:1 male to female cancer ratio. Male predominance in incidence was most marked in embryonal tumors (IRR = 1.76, 95% CI: 1.31, 2.34). The incidence rate of CNS tumors decreased with age, as the highest incidence rate was in children aged 0–4 years (5.08, 95% CI 4.60, 5.56) and the lowest in children aged 10–14 (3.70, 95% CI 3.30, 4.10). Older children aged 10–14 years had the highest proportion (35%) of benign tumors (grade I), while the youngest age group had the highest proportion (42%) of highly malignant tumors (grade IV). Most tumors located in the spinal cord and meninges were diagnosed in the age group 10–14 years.

**Fig. 1:**
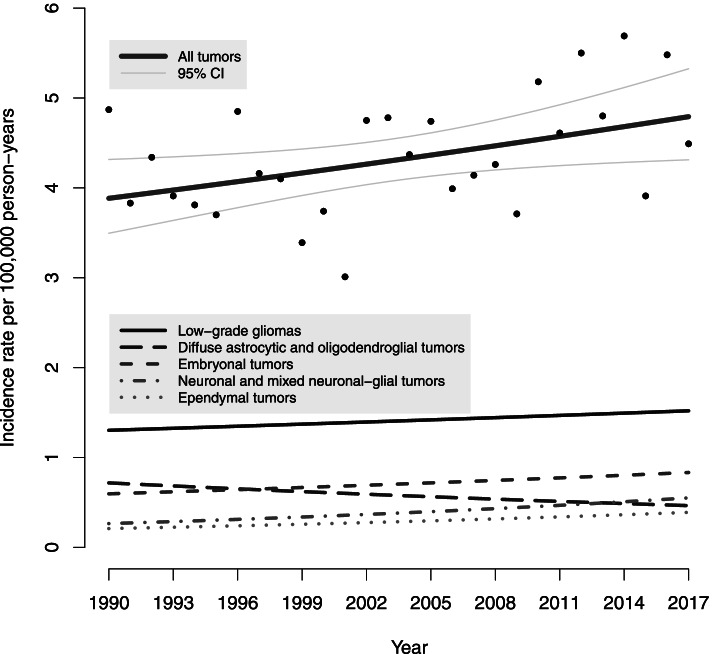
Incidence trends of pediatric CNS tumors by histology group

The total ASR increased from 4.12 (95% CI 4.03, 4.21) in 1990–1994 to 4.81 (95% CI 4.71, 4.91) in 2013–2017 (Table 3). The annual percentage change (APC) for all tumors was 0.8% per year (95% CI 0.2, 1.4). The ASR increased by 0.4% per year (95% CI -0.8, 1.5) for age group 1–4 years, by 1% per year (95% CI -0.4, 2.3) for age group 5–9 years, and by 1.1% per year (95% CI -0.3, 2.4) for age group 10–14. No major changes in incidence rate trends were observed for tumor histology groups or tumor location groups, as shown by APC values in Table 3. The ASR increased in all tumor histology groups except diffuse astrocytic and oligodendroglial tumors (Fig. 1). The incidence of benign tumors (grade I) increased by an APC of 1.0% per year (95% CI 0.1, 2.0).

**Table 3:** Incidence trends and five-year age-standardized incidence rates (ASR) per 100,000 person-years with 95% confidence intervals (CIs) of pediatric CNS tumors in Finland

	1990–2017	1990–1994		2001–2005		2013–2017	
	APC (95% CI)	N	ASR (95%)	N	ASR (95%)	N	ASR (95%)
**Total**	0.8 (0.2, 1.4)	201	4.12 (4.03, 4.21)	195	4.25 (4.15, 4.34)	216	4.81 (4.71, 4.91)
**Sex**							
Boys	0.9 (0.1, 1.8)	105	4.20 (4.07, 4.32)	117	4.99 (4.85, 5.14)	117	5.09 (4.94, 5.24)
Girls	0.7 (−0.3, 1.7)	96	4.04 (3.91, 4.17)	78	3.47 (3.35, 3.60)	99	4.52 (4.37, 4.66)
**Age group**							
0–4 years	0.4 (−0.8, 1.5)	85	5.28 (4.16, 6.41)	68	4.78 (3.65, 5.92)	82	5.60 (4.39, 6.82)
5–9 years	1.0 (−0.4, 2.3)	61	3.81 (2.86, 4.77)	66	4.30 (3.26, 5.33)	70	4.57 (3.50, 5.64)
10–14 years	1.1 (−0.3, 2.4)	55	3.37 (2.48, 4.26)	61	3.71 (2.78, 4.65)	64	4.33 (3.27, 5.39)
**Tumor grade**							
Grade I	1.0 (0.1, 2.0)	80	1.64 (1.59, 1.70)	96	2.09 (2.02, 2.15)	98	2.20 (2.13, 2.27)
Grade II	−0.5 (−2.5, 1.5)	41	0.85 (0.81, 0.89)	27	0.59 (0.56, 0.63)	26	0.57 (0.54, 0.61)
Grade III	2.6 (0.0, 5.3)	13	0.27 (0.24, 0.29)	20	0.44 (0.40, 0.47)	25	0.55 (0.51, 0.58)
Grade IV	0.7 (−0.4, 1.8)	48	0.97 (0.93, 1.02)	42	0.91 (0.87, 0.96)	45	1.00 (0.95, 1.04)
**Tumor location**							
Supratentorial	−0.9 (−2.1, 0.4)	65	1.33 (1.28, 1.38)	74	1.61 (1.55, 1.67)	50	1.11 (1.06, 1.16)
Infratentorial	0.4 (−0.8, 1.6)	86	1.76 (1.70, 1.82)	86	1.87 (1.81, 1.93)	88	1.95 (1.89, 2.02)
Cerebellum	0.1 (−1.2, 1.4)	59	1.20 (1.15, 1.25)	59	1.28 (1.23, 1.33)	55	1.22 (1.17, 1.27)
Brain stem	1.6 (−0.4, 3.7)	26	0.54 (0.51, 0.57)	26	0.57 (0.54, 0.61)	32	0.71 (0.67, 0.75)
Spinal cord	1.0 (−1.3, 3.4)	15	0.31 (0.29, 0.34)	11	0.24 (0.22, 0.26)	14	0.32 (0.29, 0.34)
Unspecified	2.5 (−2.5, 7.8)	30	0.61 (0.58, 0.65)	20	0.44 (0.41, 0.47)	58	1.29 (1.24, 1.35)
**Histology**							
Diffuse astrocytic tumors	−1.7 (−3.5, 0.1)	44	0.91 (0.87, 0.95)	33	0.72 (0.68, 0.76)	23	0.52 (0.48, 0.55)
Other astrocytic tumors	0.6 (−0.6, 1.8)	63	1.30 (1.25, 1.35)	67	1.45 (1.40, 1.51)	66	1.48 (1.42, 1.54)
Pilocytic astrocytoma	0.4 (−0.9, 1.7)	56	1.15 (1.10, 1.20)	63	1.37 (1.31, 1.42)	58	1.30 (1.24, 1.35)
Ependymal tumors	1.9 (−0.5, 4.4)	13	0.27 (0.25, 0.29)	20	0.44 (0.41, 0.47)	23	0.50 (0.47, 0.54)
Neuronal/glial tumors	1.4 (−1.3, 4.2)	13	0.27 (0.24, 0.29)	23	0.50 (0.47, 0.53)	23	0.52 (0.48, 0.55)
Embryonal tumors	1.2 (−0.1, 2.6)	34	0.68 (0.65, 0.72)	27	0.59 (0.55, 0.62)	39	0.86 (0.82, 0.90)
Medulloblastoma	1.6 (−0.4, 3.6)	22	0.45 (0.42, 0.48)	14	0.31 (0.28, 0.33)	30	0.66 (0.62, 0.70)
Other tumors	1.5 (−0.0, 3.1)	34	0.69 (0.65, 0.73)	25	0.55 (0.51, 0.58)	42	0.93 (0.89, 0.98)

## Discussion

In this nationwide register-based study, we estimated the incidence trends of pediatric CNS tumors in Finland during 1990–2017. We observed an increase of 0.8% per year in the overall incidence of pediatric CNS tumors. The average ASR increased from 4.12 in 1990–1994 to 4.81 in 2013–2017. We observed no major changes in incidence trends of tumor histology groups or tumor location groups.

We categorized CNS tumors into broad groups based on the 2016 WHO classification of CNS tumors, which incorporated molecular parameters for the first with the addition of newly recognized neoplasms and removal of a few others [9]. The FCR adopted the ICD-O-3 coding system in 2008 and retrospectively recoded the tumors previously registered based on the ICD-7. The recoding reduced the number of unspecified tumor codes such as glioma malignant NOS (code 9380), which comprised 3% of all the tumors in our dataset compared to 14% in the US and 15% in Canada [5, 6]. A proportion of glioma malignant NOS was recoded into pilocytic astrocytoma, which explains the higher proportion of pilocytic astrocytomas in our dataset (30%) compared to studies in France (22%) and the UK (21%) [7, 16]. However, the frequencies of other tumor groups in our study were comparable to the study conducted in France, which reported 14% medulloblastomas, 7% ependymomas, and 9% neuronal and mixed neuronal-glial tumors [7]. Similar to earlier studies, we observed that the incidence of CNS tumors in children declined with age and that there was a male predominance, particularly for embryonal tumors [5–7, 17].

The observed increase in trend might be explained by a true increase in cancer incidence. However, several other factors might have led to fluctuations in overall and subgroup incidence rates over time and need to be considered before accepting such interpretation. CNS tumors comprise more than 100 histological subtypes [8]. This can lead to variations in classification and registration practices between registers and over time, complicating international incidence comparisons and time trends analysis. Studies from the US, Europe, and the Nordic countries have indicated an increasing incidence of pediatric CNS tumors from the 1970s to the 1990s [3, 4, 18–21]. This rise occurred primarily in the mid-1980s due to enhanced detection and earlier diagnosis driven mainly by the introduction of MRI [4]. It is unclear whether the increasing availability and accessibility of MRI and the advent of advanced diagnostic techniques such as diffusion-weighted MRI and MR spectroscopy have continued to facilitate the detection and characterization of CNS tumors after 2000. In our study the incidence of benign tumors increased consistently throughout the study period, which might indicate that improved diagnostics had led to earlier detection of slow-growing tumors. A study from the US reported a significant increase of 0.6% per year for malignant CNS tumors and 2.3% per year for non-malignant tumors in children between 2000 and 2015 [6]. However, a Canadian study found a stable incidence during the same time period [5]. In Finland, the annual number of pediatric head MRIs increased from 4.1 per 1000 children in 2008 to 7.3 per 1000 children in 2018 [22]. Concurrently, the use of CT in pediatric imaging has been declining in Finland, similar to several other Western countries [23–26].

Compared with our results (overall incidence 4.3 per 100,000), lower incidence rates have been reported in some other Western countries such as Canada (3.8/100,00), France (3.9/100,000), and Britain (4/100,000), while a higher rate was reported in the US (5.7/100,000) [5–7, 16]. Higher incidence rates have been reported in the Nordic countries than in other European countries [27, 28]. The completeness of the Finnish Cancer Registry has been shown to be very high [11]. For childhood tumors, the completeness of FCR data was estimated as 94% for 2009–2013 with registration of benign brain tumors since its establishment in 1953 [13]. Automated reporting of histologically or cytologically confirmed cases from pathological laboratories has been practically comprehensive since the late 1980s and hence the coverage of pathologically verified diagnoses is nearly complete. Clinical notifications, the other source of case ascertainment from hospitals, on the other hand, have been based on manual forms until recently (a simplified electronic format for clinical notifications was introduced in 2020) and their number relative to pathological notifications has been declining over time [29]. During the past few decades, cross-checking with both hospital discharge register and cause of death database as independent sources of information have enabled the identification of any missed cases [11]. The comprehensive use of unique personal identifiers also allows the elimination of duplicate cases. The potential impact of any changes in coverage of cancer on the incidence of pediatric CNS tumors over time is difficult to evaluate. Clinical notifications are essential for compiling information on tumor stage, localization, and treatment, but information crucial for incidence analysis (date of diagnosis, histological type, and demographics) is available from the electronic reports by the pathology laboratories. Any incompleteness is, therefore, most likely to affect mainly the earliest part of the study period and could accentuate an increasing trend through case undercount.

Established risk factors for pediatric CNS tumors remain limited to familial cancer syndromes and high doses of ionizing radiation [8]. However, it is unlikely that these factors have contributed to the increasing incidence of pediatric CNS tumors. The use of radiation therapy has declined over time in most pediatric cancer categories [30]. Exposure to radiation from CT imaging in children has been linked to a higher risk of malignancy such as leukemia and brain cancer [31–35]. Nevertheless, CT use in children started to decline in Finland after 2002 with increasing awareness of radiation-related risks and reliance on other imaging modalities [23]. Familial cancer syndromes such as tuberous sclerosis and neurofibromatosis can carry very high risks of brain tumors, but their prevalence is very low and stable and cannot explain the observed changes in incidence. There are several suspected risk factors for pediatric CNS tumors that are still the focus of research in the field. Growing evidence indicates a positive association with maternal dietary supplements, advanced parental age, pesticide exposure, birth weight, and birth defects [8]. As the etiology of brain cancer is still largely unknown, we also need to consider the possibility of other unknown risk factors which might have affected the incidence of pediatric CNS tumors. If the observed increase is to be explained by a specific exposure, it would be expected to be increasing gradually over several decades.

A limitation in our study is the small number of cases in some histology and topography groups, such as germ cell tumors and spinal cord tumors. Therefore, we were unable to provide a detailed analysis of incidence trends for these groups. Moreover, the statistical power to show differences in incidence trends between tumor subgroups was limited. However, our 27-year study period allowed the aggregation of relatively large numbers in the main CNS tumor groups. In addition, the centralized cancer care and quality of cancer registration in Finland contributed to the completeness of data and comparability across the entire population. Moreover, benign CNS tumors were included constantly throughout the whole study period.

In conclusion, we found a minor increase in the incidence of pediatric CNS tumors in Finland between 1990 and 2017. Although changes in registration practices and enhanced detection by improved and more available diagnostics could have driven the trend, a true increase in CNS cancer incidence and the contribution of environmental risk factors cannot be ruled out. Thus, continuous monitoring of incidence trends and further research into the etiology of childhood CNS tumors are warranted. One suggestion is a larger study with pooled Nordic data and a more detailed classification.

## Data Availability

The datasets used for analyses in the current study are available in the Zenodo repository at 10.5281/zenodo.5789368

## Abbreviations

APC: Average parentage change
ASR: Age-standardized incidence rate
CI: Confidence interval
CNS: Central nervous system
CT: Computed tomography
FCR: Finnish cancer registry
ICD: International classification of diseases
IRR: Incidence rate ratio
MRI: Magnetic resonance imaging
NOS: Not otherwise specified
